# Differential Effects of Marimastat and Prinomastat on the Metalloprotease Activity of Various Snake Venoms

**DOI:** 10.3390/toxins17120571

**Published:** 2025-11-26

**Authors:** Mahtab Khatibi, José R. Almeida, Soheil Gilabadi, David Ramírez, Paulina Valenzuela-Hormazábal, Ketan Patel, Sakthivel Vaiyapuri

**Affiliations:** 1 School of Pharmacy, University of Reading, Reading RG6 6UB, UK; m.khatibi@student.reading.ac.uk (M.K.); rafael.dealmeida@ikiam.edu.ec (J.R.A.); s.gilabadi@pgr.reading.ac.uk (S.G.); 2 Biomolecules Discovery Group, Universidad Regional Amazónica Ikiam, Km 7 Via Muyuna, Tena 150150, Ecuador; 3 Departamento de Farmacología, Facultad de Ciencias Biológicas, Universidad de Concepción, Concepción 4030000, Chile; dramirezs@udec.cl (D.R.); paulinvalenzuela@udec.cl (P.V.-H.); 4 School of Biological Sciences, University of Reading, Reading RG6 6UB, UK; ketan.patel@reading.ac.uk

**Keywords:** snake venom, metalloprotease, marimastat, prinomastat, elapids, cobra, vipers, viperid

## Abstract

Snakebite envenoming is a neglected tropical disease, responsible for approximately 140,000 deaths globally each year. Vipers and elapid snakes represent the most significant snake families in medical contexts, exhibiting a variety of venom components and clinical effects in bite victims. Metalloproteases, a primary component of venoms, are mainly accountable for haemotoxic and myotoxic effects. Although predominantly found in viper venoms, these enzymes are also present in varying levels in elapid snake venoms. Marimastat and prinomastat are matrix metalloprotease inhibitors initially developed as cancer therapies. Recently, extensive research has focused on these inhibitors to neutralise venom metalloproteases. However, their effects on different viper and elapid snake venoms remain unclear. Here, we report the sensitivity of seven elapid venoms (specifically, cobras) and 12 viper venoms to marimastat and prinomastat, utilising selective in vitro experiments and molecular docking analyses performed using representative metalloprotease (VAP2, a viper metalloprotease from the venom of *Crotalus atrox* and an elapid metalloprotease from the venom of *Naja atra*) structures. Both compounds inhibited the metalloprotease, fibrinogenolytic, and caseinolytic activities of most viper venoms. While prinomastat displayed prominent inhibitory effects on cobra venoms in these assays, marimastat demonstrated limited inhibitory effects on these venoms. These findings illustrate the role of matrix metalloprotease inhibitors in modulating metalloprotease activities across a range of viper and cobra venoms. Collectively, this study establishes the differential effects of marimastat and prinomastat on various levels of metalloproteases present in viper and elapid venoms. This will enhance understanding of the abundance of metalloproteases in snake venoms and their sensitivity to different matrix metalloprotease inhibitors.

## 1. Introduction

Snake venoms are intricate mixtures of proteins that induce various toxic effects. The significant variability in snake venom constituents results in differing clinical envenomation outcomes, ranging from local tissue damage to potentially fatal systemic effects [1]. Snake venoms primarily consist of enzymatic and non-enzymatic proteins [2]. The enzymatic components include snake venom metalloproteases (SVMPs), snake venom serine proteases (SVSPs), and phospholipases A_2_ (PLA_2_s). The non-enzymatic components of venom primarily consist of three-finger toxins (3FTXs), C-type lectin-like proteins, and disintegrins [3]. SVMPs are zinc-dependent enzymes with a molecular mass ranging from approximately 20 kDa to 100 kDa. SVMPs are mainly responsible for venom-induced haemotoxicity, local tissue necrosis, renal failure, and specific systemic complications. They are predominantly found in viper venoms, although low quantities can be present in elapid snake (e.g., cobras) venoms [3,4,5]. SVMPs are mainly classified into three categories: PI, which contains a metalloprotease domain; PII, which possesses a disintegrin domain alongside the metalloprotease domain; and P-III, which incorporates metalloprotease, disintegrin-like, and cystine-rich domains [4,6]. SVMPs exhibit a wide range of biological activities, including the degradation of collagen, fibrinogen, and other constituents of the basement membrane, which can lead to muscle damage surrounding the bite site and bleeding complications [3,4,5]. Currently used antivenoms are largely ineffective in addressing local envenomation effects due to their inability to reach the affected tissues and neutralise tissue-bound venoms, likely due to their large molecular weights and obstructed blood capillaries [7,8]. Hence, the urgent development of small-molecule drugs that can counteract the metalloprotease activities in venoms and prevent local tissue necrosis is essential [5].

Marimastat and prinomastat are synthetic peptidomimetic, small-molecule drugs initially developed to inhibit matrix metalloproteases (MMPs) and aid in cancer treatment. Marimastat is a low-molecular-weight, orally bioavailable molecule designed to provide broad-spectrum, non-selective inhibition of MMPs by mimicking the cleavage site of their substrates and binding to the Zn^2+^ ion at the active site of these enzymes [5,6,9]. Prinomastat is also a potent, orally administered, but selective inhibitor of MMPs-2, -3, -9, -13, and -14 [10,11]. Both molecules failed in clinical trials for cancer due to the side effects associated with long-term use [9,11]. However, these drugs have recently attracted the attention of venom researchers to repurpose them to counteract the actions of SVMPs in various venoms. Recent in vivo venom-neutralisation studies demonstrated that using a combination of marimastat with a PLA_2_ inhibitor (varespladib) can mitigate the lethal complications, such as haemorrhage and coagulopathic effects, of several medically important viper venoms from different regions and protect the mice from venom-induced lethality [12]. Nevertheless, the sensitivity of various viper and elapid venoms to these drugs has yet to be fully elucidated.

Here, we report the effects of marimastat and prinomastat on SVMP-mediated activities in vitro in different viper and cobra venoms, thereby demonstrating their differential actions in such venoms. These data provide a better understanding of the levels of SVMPs in viper and elapid venoms and their sensitivity to marimastat and prinomastat, guiding future development of these molecules for treating snakebites.

## 2. Results

### 2.1. Viper and Elapid Venoms Exhibit Distinct Protein Profiles

**Figure 1:**
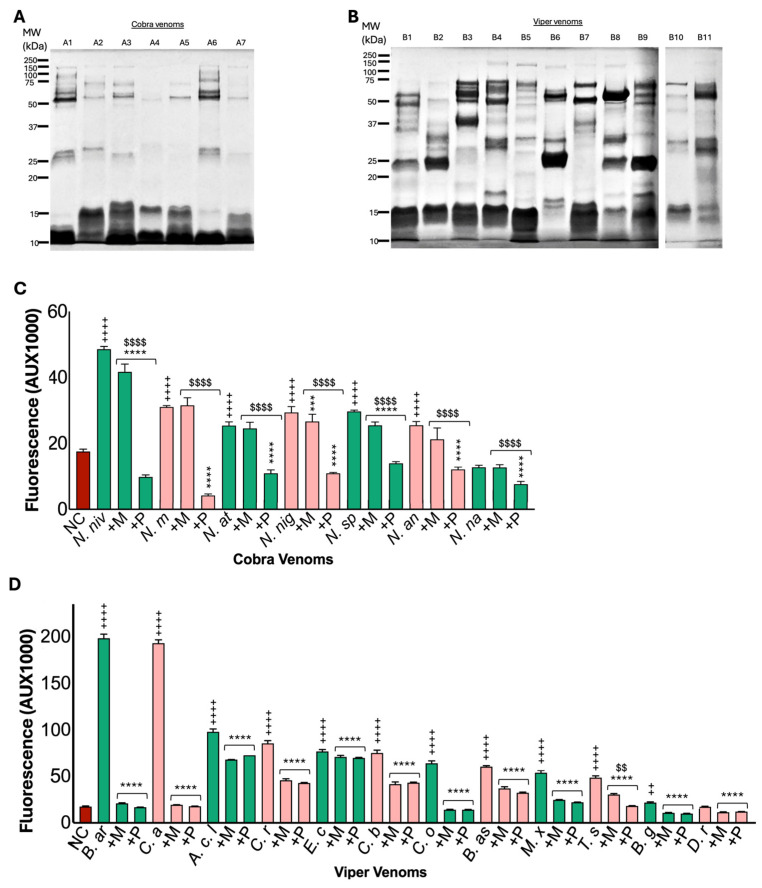
Electrophoretic and enzymatic profiles of cobra and viper venoms. Images of Coomassie blue-stained SDS-PAGE gels (12%) showing the protein profiles of cobra (**A**) and viper (**B**) venoms (60 μg in each lane). In the gel, the cobra venoms are included as *Naja nivea* (A1), *N. melanoleuca* (A2), *N. atra* (A3), *N. nigricollis* (A4), *N. sputatrix* (A5), *N. annulifera* (A6), and *N. naja* (A7). The viper venoms are included as *Echis carinatus* (B1), *Agkistrodon contortrix laticinctus* (B2), *Montivipera xanthina* (B3), *Trimeresurus stejnegeri* (B4), *Daboia russelii* (B5), *Crotalus basiliscus* (B6), *Bitis gabonica* (B7), *Crotalus atrox* (B8), *Bitis arietans* (B9), *Bothrops asper* (B10) and *Causus rhombeatus* (B11). Due to the limited availability of *Crotalus oreganus* venom, SDS-PAGE analysis could not be performed for this sample. MW—represents the protein molecular weight marker. Metalloprotease activities of 7 cobra (100 μg/mL each) (**C**) and 12 viper (50 μg/mL each) (**D**) venoms were quantified in the absence and presence of metalloprotease inhibitors [100 µM marimastat (M) or prinomastat (P)] using DQ-gelatin as a substrate by spectrofluorimetry. The substrate in the absence of the venom was used as the negative control (NC). ^+^ represents difference between NC and venom; * represents difference between respective venom and inhibitor (M/P); and ^$^ represents difference between M and P. Two-way ANOVA revealed significant effects of species, treatment, and their interaction in both venom groups (cobra: species F(7,110) = 448.12, *p* < 0.001; treatment F(2,110) = 2610.06, *p* < 0.001; interaction F(12,110) = 148.46, *p* < 0.001; viper: species F(12,185) = 5063.42, *p* < 0.001; treatment F(2,185) = 25,210.90, *p* < 0.001; interaction F(22,185) = 3257.14, *p* < 0.001). Shapiro–Wilk tests confirmed approximate normality of residuals, while Levene’s tests indicated variance heterogeneity (*p* < 0.001); analyses remained robust due to balanced designs. Tukey’s post-hoc analyses showed that both inhibitors significantly reduced MP activity compared with venom-alone groups (*p* < 0.001). Data represent mean ± S.D. (*n* = 6), and the *p* values (**** *p* < 0.0001, *** *p* < 0.001 and ** *p* < 0.01; the same *p* values apply to other symbols) shown are calculated using two-way ANOVA followed by Tukey’s post-hoc test.

To determine the protein profiles of selected cobra (*Naja annulifera*, *Naja atra*, *Naja melanoleuca*, *Naja naja*, *Naja nigricollis*, *Naja nivea*, and *Naja sputatrix*) and viper (*Agkistrodon contortrix laticinctus*, *Bitis arietans*, *Bitis gabonica*, *Bothrops asper*, *Causus rhombeatus*, *Crotalus atrox*, *Crotalus basiliscus*, *Daboia russelii*, *Echis carinatus*, *Montivipera xanthina*, and *Trimeresurus stejnegeri*) venoms, we analysed them using SDS-PAGE. The venoms of the cobra (Figure 1A) and viper (Figure 1B) exhibited diverse protein profiles, comprising various proteins with different molecular weights. In all the cobra venoms, abundant proteins were detected in the 10–18 kDa molecular weight range, with very few low-abundance proteins found at higher molecular weight ranges (25–150 kDa). Both *N. annulifera* and *N. nivea* venoms displayed two high-intensity bands at approximately 25 and 50 kDa. In viper venoms, except *C. basiliscus* (20 to 75 kDa), large and abundant proteins were observed across the 10–75 kDa molecular weight range.

### 2.2. Most Cobra and Viper Venoms Exhibit Metalloprotease Activity

To determine the metalloprotease activities of the venoms and their sensitivity to marimastat and prinomastat, DQ-gelatin, a fluorogenic substrate, was employed. Cobra venoms (100 μg/mL) exhibited low metalloprotease activity compared to 50 μg/mL of viper venoms (Figure 1C). Notably, *N. nivea* venom demonstrated the highest metalloprotease activity among the cobra venoms, and *N. naja* venom exhibited markedly low metalloprotease activity despite the higher concentrations of venom used in the assay. Conversely, viper venoms (50 μg/mL) revealed high levels of metalloprotease activities. Specifically, *B. arietans* and *C. atrox* displayed twofold higher activity than that of the other viper venoms at the same concentrations (Figure 1D).

To investigate the ability of marimastat and prinomastat to inhibit the metalloprotease activities of the venoms, the assay was conducted both in the presence and absence of these inhibitors. The metalloprotease activities of most cobra venoms were inhibited by prinomastat (100 μM) (Figure 1C). However, marimastat (100 μM) only inhibited specific cobra venoms, although the degree of this inhibition was significantly lower with marimastat compared to prinomastat at the same concentrations. For instance, the metalloprotease activity of *N. melanoleuca* and *N. atra* was strongly inhibited by prinomastat, whereas marimastat did not induce any significant inhibition in these venoms. Nonetheless, both inhibitors significantly reduced the metalloprotease activities of all selected viper venoms at similar levels (Figure 1D). These data suggest differential levels of metalloprotease activities in cobra and viper venoms, as well as varying sensitivities of these venoms to marimastat and prinomastat.

### 2.3. Marimastat Does Not Effectively Inhibit the Metalloprotease Activities of Cobra Venoms

**Figure 2:**
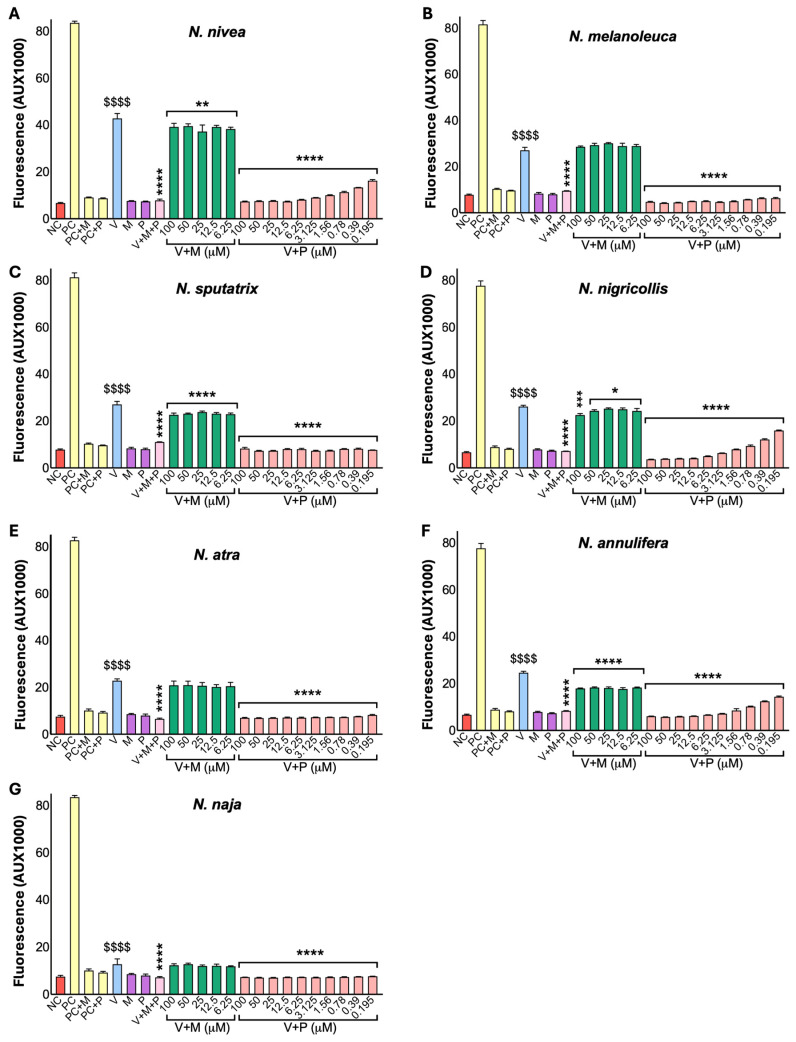
Inhibitory effects of marimastat and prinomastat on the metalloprotease activity of cobra venoms. Metalloprotease activities of *N. nivea* (**A**), *N. melanoleuca* (**B**), *N. sputatrix* (**C**), *N. nigricollis* (**D**), *N. atra* (**E**), *N. annulifera* (**F**), and *N. naja* (**G**) in the presence and absence of different concentrations of marimastat (M) and prinomastat (P) were assessed for 90 min using fluorogenic DQ-gelatin as a substrate by spectrofluorimetry. The negative control (NC) was included without any venom. All concentrations of inhibitors were compared with the venom alone control (V). PC indicates the positive control (50 μg/mL of *C. atrox* venom). ^$^ denotes significant differences compared to the NC, and * indicates significant differences compared to V. Data represent mean ± S.D. (*n* = 6). The *p*-values (**** *p* < 0.0001, *** *p* < 0.001, ** *p* < 0.01 and * *p* < 0.05; the same *p* values apply to other symbols) shown were calculated by one-way ANOVA with multiple comparisons with uncorrected Fisher’s LSD using GraphPad Prism.

**Figure 3:**
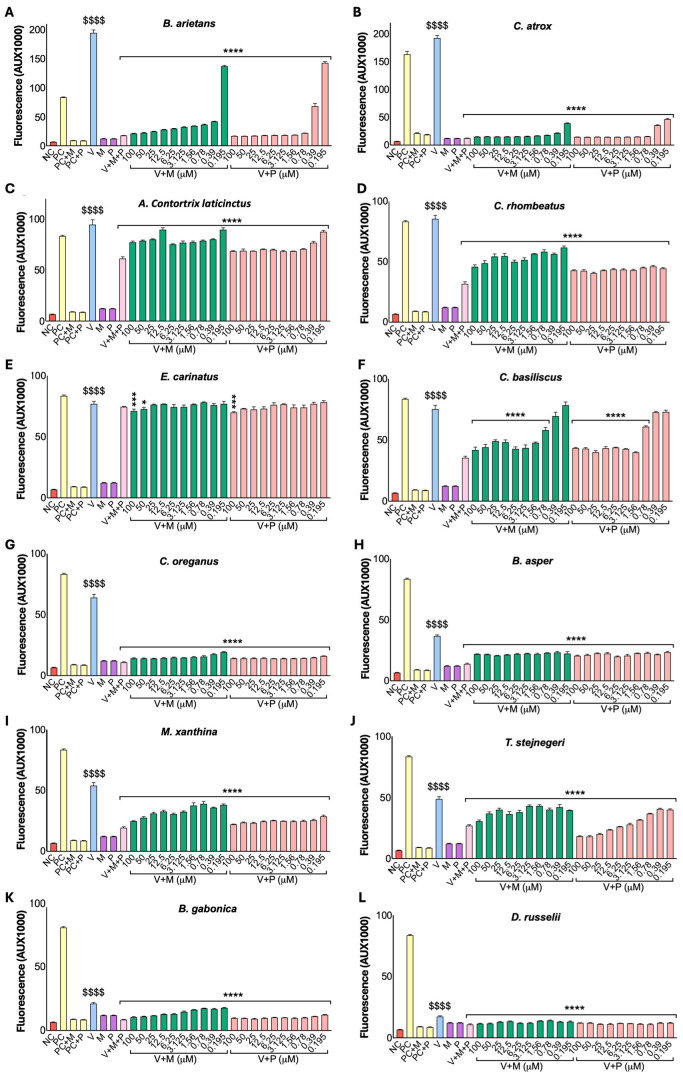
Inhibitory effects of marimastat and prinomastat on the metalloprotease activity of viper venoms. Metalloprotease activities of *B. arietans* (**A**), *C. atrox* (**B**), *A. c. laticinctus* (**C**), *C. rhombeatus* (**D**), *E. carinatus* (**E**), *C. basiliscus* (**F**), *C. oreganus* (**G**), *B. asper* (**H**), *M. xanthina* (**I**), *T. stejnegeri* (**J**), *B. gabonica* (**K**) and *D. russelii* (**L**) with different concentrations of marimastat (M) and prinomastat (P) were tested using fluorogenic DQ-gelatin as substrate for 90 min by spectrofluorimetry. The negative control (NC) without venom was included. All concentrations of inhibitors were compared with the venom alone control (V). PC represents the positive control (50 μg/mL of *C. atrox* venom). ^$^ shows significant differences compared to the NC, and * indicates significant differences compared to V. Data represent mean ± S.D. (n = 6). The *p*-values (**** *p* < 0.0001, *** *p* < 0.001 and * *p* < 0.05; the same *p*-values apply to other symbols) shown were calculated by one-way ANOVA with multiple comparisons with uncorrected Fisher’s LSD using GraphPad Prism.

To further analyse the level of inhibition on metalloprotease activities of all selected cobra and viper venoms, several concentrations (0.195 to 100 µM) of marimastat and prinomastat were tested with these venoms. Both marimastat and prinomastat caused significant inhibition of the metalloprotease activities of most cobra venoms (Figure 2), except in the venoms of *N. melanoleuca*, *N. atra* and *N. naja*. However, the inhibition achieved by marimastat was significantly lower compared to that of prinomastat in cobra venoms. Prinomastat exhibited a dose-dependent inhibition in most venoms. However, its lowest dose (0.195 µM) appears to be sufficient to cause maximal inhibition of metalloprotease activities in the venoms of *N. sputatrix*, *N. atra*, and *N. naja*, as no significant activity was observed compared with the negative control. Most viper venoms, on the other hand, displayed high sensitivity to both marimastat and prinomastat (Figure 3). The venoms of *A. c. laticinctus* and *E. carinatus* showed minimal inhibition with either of the drugs, even at higher concentrations. Venoms from *Causus rhombeatus*, *Crotalus basiliscus*, *M. xanthina*, and *T. stejnegeri* were only partially inhibited by both marimastat and prinomastat. However, both inhibitors substantially inhibited the metalloprotease activity of other viper venoms in a dose-dependent manner.

### 2.4. Viper Venoms Exhibit Greater Proteolytic Activities than Cobra Venoms

**Figure 4:**
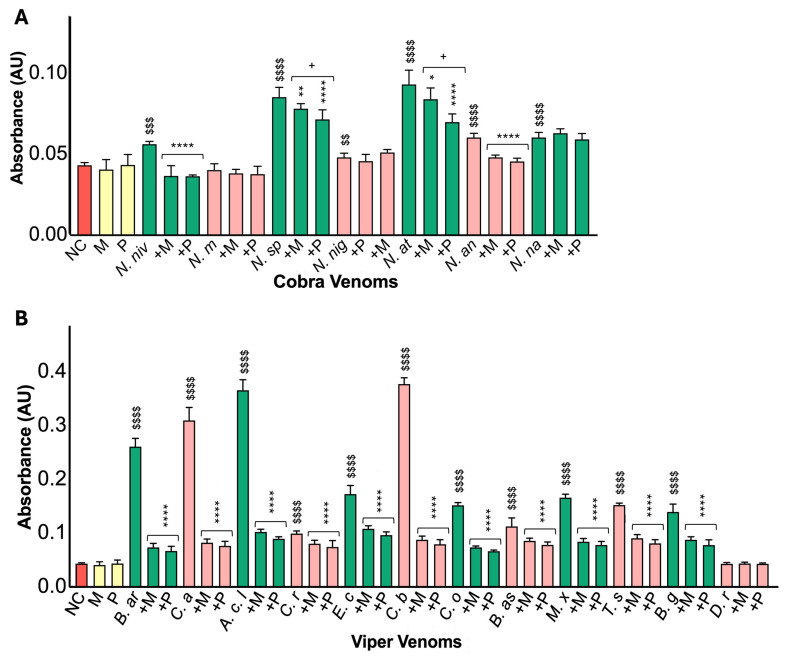
Impact of marimastat and prinomastat on the caseinolytic activities of cobra and viper venoms. The caseinolytic activities of cobra (**A**) and viper (**B**) venoms were analysed in the presence and absence of 100 µM marimastat (M) or prinomastat (P). Cobra (100 µg/mL) and viper (50 µg/mL) venoms were incubated with azocasein at 37 °C for 90 min. Samples were then centrifuged for 5 min at 8000 rpm after the addition of 5% (*v*/*v*) trichloroacetic acid (TCA) to remove non-digested proteins. The protein content of the supernatant was measured by spectrofluorimetry at 440 nm. In the negative control (NC), PBS was replaced with venom. ^$^ indicates significant differences relative to the NC, * denotes significant differences between inhibitor-treated samples and their corresponding venom-alone (V) samples and ^+^ shows differences between M and P. Two-way ANOVA revealed significant main effects of species, treatment, and their interaction in both venom groups (cobra: species F(7,120) = 251.58, *p* < 0.001; treatment F(2,120) = 57.55, *p* < 0.001; interaction F(14,120) = 8.71, *p* < 0.001; viper: species F(12,195) = 424.14, *p* < 0.001; treatment F(2,195) = 3569.14, *p* < 0.001; interaction F(24,195) = 248.36, *p* < 0.001). Shapiro–Wilk tests confirmed the approximate normality of residuals, while Levene’s tests indicated variance heterogeneity (*p* < 0.001); however, the results were considered robust due to balanced group sizes. Tukey’s post-hoc analyses showed that both M and P significantly reduced caseinolytic activity compared with venom-alone controls (*p* < 0.001). Data are presented as mean ± SEM (n = 6). The *p*-values (**** *p* < 0.0001, *** *p* < 0.001, ** *p* < 0.01 and * *p* < 0.05; the same *p*-values apply to other symbols) shown are calculated using two-way ANOVA followed by Tukey’s post-hoc test in SPSS.

To assess the proteolytic activities of cobra and viper venoms, the caseinolytic assay was employed. The results indicated that viper venoms (50 µg/mL) exhibited considerably higher caseinolytic activities compared to cobra venoms (100 µg/mL) (Figure 4). All cobra venoms, except *N. melanoleuca*, demonstrated significant caseinolytic activities relative to the negative control (Figure 4A). *N. melanoleuca* venom displayed the least or no activity among the cobra venoms. Both marimastat and prinomastat markedly inhibited the proteolytic activities of *N. nivea*, *N. sputatrix*, *N. atra*, and *N. annulifera*. Notably, prinomastat resulted in more potent inhibition in *N. sputatrix* and *N. atra*. There was no significant inhibition from either inhibitor in *N. nigricollis*, although the activity was low. Conversely, all viper venoms, excluding *D. russelii*, exhibited significant proteolytic activity compared to the negative control (Figure 4B). *B. arietans*, *C. atrox*, *A. c. laticinctus*, and *C. basiliscus* showed nearly eight times greater activities compared to the negative control. The remaining viper venoms (except *D. russelli*) demonstrated approximately two to four times higher protease activities relative to the negative control. In all viper venoms (except *D. russelli*, where no activity was observed), both inhibitors successfully inhibited the caseinolytic activities at comparable levels, despite the lower concentrations (50 µg/mL) of venom used in the assay.

### 2.5. Selective Cobra and All Viper Venoms Exhibit Fibrinogenolytic Activities

**Figure 5:**
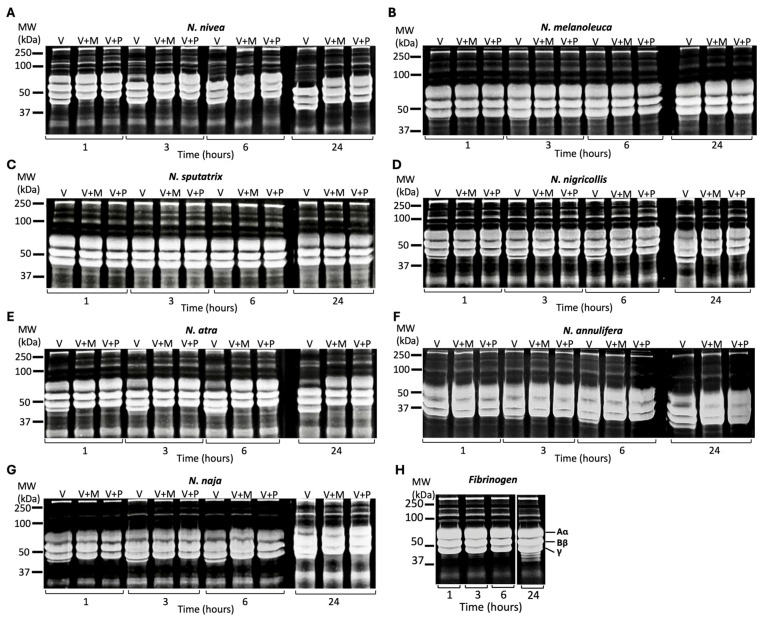
Effects of marimastat and prinomastat on the fibrinogenolytic activities of cobra venoms. SDS-PAGE gels (12%) display the fibrinogenolytic activities of the cobra venoms (100 µg/mL) following their incubation with human fibrinogen (1 mg/mL) at 37 °C for 1, 3, 6 and 24 h: *N. nivea* (**A**), *N. melanoleuca* (**B**), *N. sputatrix* (**C**), *N. nigricollis* (**D**), *N. atra* (**E**), *N. annulifera* (**F**) and *N. naja* (**G**). The fibrinogenolytic activities of cobra venoms have been assessed alone (V) and with marimastat (V + M) or with prinomastat (V + P) by collecting samples at 1, 3, 6 and 24 h. All samples were analysed through SDS-PAGE and Coomassie staining (gels were inverted to better visualise the digested bands) for the visualisation of protein bands. (**H**) Fibrinogen incubated without venom was used as a control. MW represents the molecular weight marker.

To analyse the fibrinogenolytic activities of the venoms, human fibrinogen was incubated with all the venoms in the presence and absence of 100 µM marimastat or prinomastat, and the samples were analysed at various time points. In cobra venoms (Figure 5), *N. nivea*, *N. atra*, and *N. naja* degraded the α chain of fibrinogen after 1 h of incubation, as shown in the reduced quantity of the α chain with increasing incubation time compared to the venom with inhibitors and the negative control (Figure 5H). In all cobra venoms, the β chain of fibrinogen was not degraded at any time point. Among all cobra venoms, *N. nivea*, *N. atra*, *N. naja*, and *N. nigricollis* showed the most fibrinogenolytic activity. These venoms also produced various digested products, which appeared as multiple faint bands between 15 and 25 kDa on the gels and became stronger as incubation time progressed (Figure S1). In cobra venoms, *N. sputatrix* exhibited no fibrinogenolytic activity, as it displayed the same bands as the venom with inhibitors and the negative control at all time points. *N. annulifera* demonstrated slight fibrinogenolytic activity only at 24 h, as its α band began to fade. In all cobra venoms that exhibited fibrinogenolytic activity, both marimastat and prinomastat completely inhibited this activity at nearly all time points, indicating that both inhibitors affect the cobra venoms-induced fibrinogenolytic activities to the same extent.

**Figure 6:**
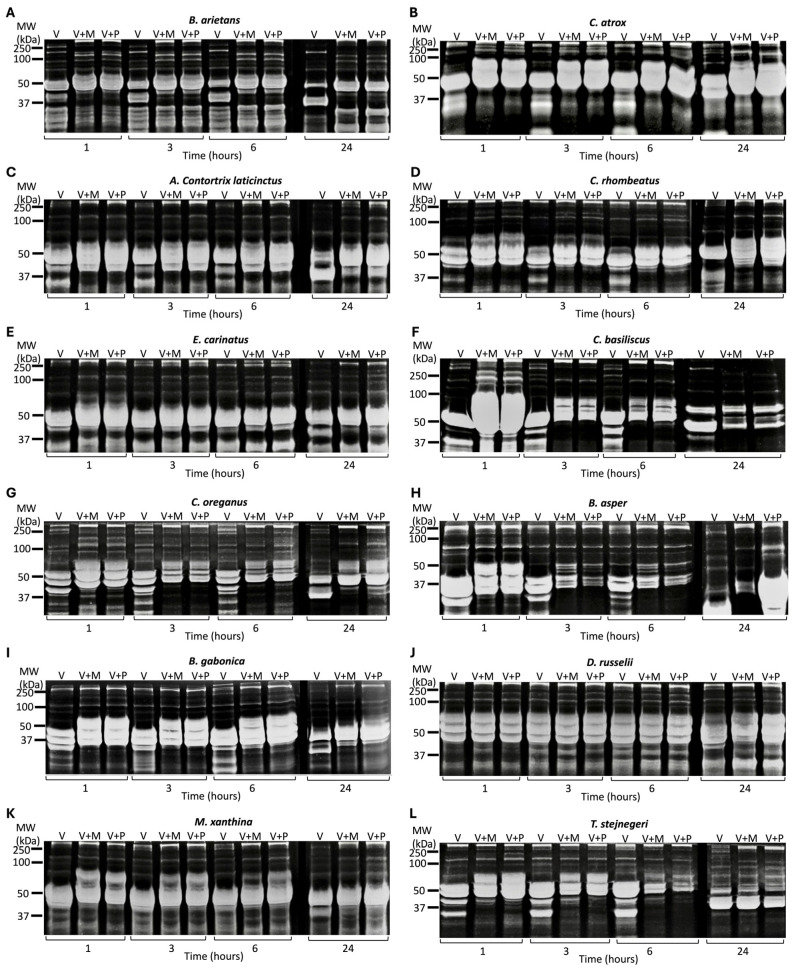
Effects of marimastat and prinomastat on the fibrinogenolytic activities of viper venoms. SDS-PAGE gels (12% gel) display the fibrinogenolytic activities of the viper venoms (50 µg/mL) following their incubation with human fibrinogen (1 mg/mL) at 37 °C for 1, 3, 6 and 24 h. *B. arietans* (**A**), *C. atrox* (**B**), *A. c. laticinctus* (**C**), *C. rhombeatus* (**D**), *E. carinatus* (**E**), *C. basiliscus* (**F**), *C. oreganus* (**G**), *B. asper* (**H**), *B. gabonica* (**I**), *D. russelii* (**J**), *M. xanthina* (**K**) and *T. stejnegeri* (**L**). The fibrinogenolytic activities of viper venoms were assessed alone (V), and with/without marimastat (V + M) or with prinomastat (V + P). These samples were analysed through incubation with human fibrinogen and collection at various time points (1, 3, 6 and 24 h). All samples were assessed through SDS-PAGE and Coomassie staining (gels were inverted to better visualise the digested bands) for the visualisation of protein bands. MW represents the molecular weight marker.

All viper venoms (50 µg/mL) were incubated with and without marimastat or prinomastat, and the samples were analysed at 1, 3, 6 and 24 h. All the venoms except *D. russelii* began digesting the α chain of fibrinogen 1 h after incubation (Figure 6). *C. basiliscus*, *C. oreganus*, and *T. stejnegeri* initiated the digestion of the β chain of fibrinogen after 24 h of incubation. In contrast, *C. rhombeatus* commenced degradation of the β chain after 6 h. *B. arietans* started the digestion of the α and β chains simultaneously, and the γ chain after 24 h of incubation. *B. asper* also began digesting the γ chain after 24 h. *D. russelii* exhibited no obvious fibrinogenolytic activity until 24 h, at which point it began degrading the α chain of fibrinogen. In *C. atrox*, *C. oreganus*, and *B. gabonica*, both marimastat and prinomastat inhibited the fibrinogenolytic activities of the venoms. However, in *A. c. laticinctus*, *C. rhombeatus*, *E. carinatus*, *C. basiliscus*, and *T. stejnegeri*, these inhibitors did not affect the digestion of the α chain of fibrinogen after 3 h of incubation. This could be due to the high venom activity and the limited amount of inhibitors.

### 2.6. Marimastat and Prinomastat Show Significant Affinities Toward the Venom Metalloproteases

**Figure 7:**
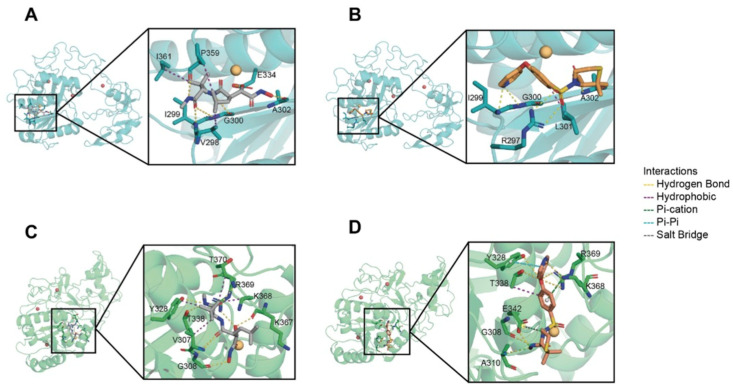
Docking analysis of SVMPs with marimastat and prinomastat. (**A**) The interactions of VAP2 (blue) with marimastat (grey) (pose 3) and prinomastat (orange) (pose 2) (**B**) were analysed. Similarly, the interactions of an elapid snake venom metalloprotease (green) with marimastat (grey) (pose 5) (**C**), and prinomastat (orange) (pose 1) (**D**) were analysed. The interactions are represented as follows: hydrogen bonds (yellow line), hydrophobic interactions (magenta line), pi-cation interactions (green line), pi-pi interactions (cyan line), and salt bridges (grey line). Zinc and calcium ions are shown in yellow and red, respectively, in the images.

To predict the interactions between marimastat or prinomastat with venom metalloproteases, molecular docking analyses were performed. VAP2, an SVMP from the venom of *C. atrox* and an elapid SVMP from the venom of *N. atra* were used to dock with marimastat and prinomastat. The docking parameters were analysed by calculating the free binding energy (ΔGBind) using the MM-GBSA method. The analyses revealed significant interactions between VAP2 and marimastat (Figure 7A) and prinomastat (Figure 7B), as well as an elapid SVMP and marimastat (Figure 7C) and prinomastat (Figure 7D). The results indicate that these drugs exhibit favourable docking scores and negative ΔGBind values, suggesting favourable binding between these molecules. The results showed excellent stability in the VAP2-marimastat and elapid SVMP-marimastat complexes throughout the 1 μs trajectory, as evidenced by the root mean square deviation (RMSD) values of the protein backbones. The RMSD values remained low and constant, suggesting that the interactions between marimastat and both proteins are robust and persistent during the simulation. In terms of ligands, the RMSD values for the protein backbones remained stable across all complexes, indicating stable interactions with the proteins. For marimastat (pose 3) and marimastat (pose 5), the RMSD values of the ligands were low, suggesting a stable interaction with the system. In contrast, for prinomastat (pose 1) and (pose 2), the RMSD values of the ligands fluctuated between 2 and 5 Å; however, the overall stability of the system was maintained. These results provide detailed insights into the conformational stability and dynamic interactions between the inhibitors and proteins.

During the 1 μs molecular dynamics (MD) simulation, we analysed the interaction frequencies of marimastat and prinomastat complexes with the same proteins. Our focus was on residues that displayed interaction frequencies higher than 20% along the trajectory. In the VAP2-marimastat complex, residues R297, G300, A302, E334, and N342 exhibited significant participation in hydrogen bonding, hydrophobic, and water-bridging interactions. Conversely, in the VAP2-prinomastat complex, residues V304 and R312 were pivotal for hydrogen bonding interactions, with a minor but significant contribution from water bridges in L301, K311, and H337. For the elapid SVMP-marimastat complex, residues G308, V307, and R369 demonstrated hydrogen bonding and water-bridging interactions. A higher frequency was noted in G308 for hydrogen bonding interactions, while R369 and T306 established persistent water-bridge interactions throughout the simulation. Finally, in the elapid SVMP-prinomastat complex, residue R369 was particularly relevant, exhibiting a combination of π-cation and water-bridging interactions. Additionally, T306 and G308 contributed to hydrogen bonding interactions, whereas V307 and T338 displayed hydrophobic interactions. These interaction profiles underscore the significance of water-bridging and hydrogen bonding interactions in the stability of the complexes, while π-cation and π-π interactions play a critical role in the interaction of prinomastat with specific protein residues. These findings suggest that both marimastat and prinomastat exhibit significant affinities towards the target residues in their respective protein complexes.

## 3. Discussion

A wide range of venom metalloproteases, along with significant variations among different species, results in diverse clinical manifestations with varying underlying molecular mechanisms [7,8]. Their abundance and roles are better understood in viper venoms than in elapid snake venoms, which are reported to contain a smaller quantity of such enzymes [1]. Venom protein profiles from both vipers and elapid snakes are extensively documented in the literature. Generally, viper venoms are recognised for containing proteins across a broader molecular weight range. In contrast, elapid snake venoms primarily comprise low-molecular-weight proteins, with only a small proportion of proteins in the higher molecular weight range [3,13]. Consistent with these findings, we observed distinct protein bands across all molecular weights for the viper venoms analysed in this study. Conversely, cobra venoms appear to exhibit a high abundance of protein bands ranging from 10 to 18 kDa, with few proteins identified at higher molecular weights. Given that metalloproteases, particularly PIII, are usually found around 60 kDa [4], the elevated level of metalloprotease activity corresponds with their prevalence in viper venoms. The increased level of metalloprotease activities in vipers compared to cobra venoms aligns with the enhanced haemotoxicity and tissue damage following viper bites. Based on our results, *N. nivea* demonstrates the highest amount of metalloprotease activity among all cobra venoms. This observation is corroborated by a study indicating the relative abundance of metalloproteases as 6.79% in *N. nivea* [7]. In comparison, the amounts of metalloproteases in *N. nigricollis*, *N. sputatrix*, and *N. atra* are approximately 2.4%, 1.3%, and 1.6%, respectively, all of which are lower than that of *N. nivea* [14]. In another study, the percentage of metalloproteases in viper venoms was reported as 56.6% in *E. carinatus*, 41% in *B. asper*, 38.5% in *B. arietans*, 30% in *B. gabonica*, and 25% in *D. russelii*, all higher than those in cobra venoms [13]. These results highlight the significant differences in metalloprotease compositions in viper and cobra venoms.

Prinomastat and marimastat are both MMP inhibitors originally developed to target tumour growth and metastasis [5,10,15]. While marimastat exerts broad-spectrum effects against all MMPs, prinomastat exhibits narrow inhibitory effects against specific MMPs. Both drugs were discontinued due to their long-term side effects and the lack of desired efficacy [9,11]. Since snakebite is an acute issue that typically does not require long-term treatment, the impact of these molecules on treating venom-induced effects has been assessed in recent years [12]. Here, we report the sensitivity of a range of viper and elapid snake venoms to these drugs. In viper venoms, where more metalloproteases are present, both marimastat and prinomastat strongly inhibited their activities. However, in cobra venoms, where fewer metalloproteases and low activity are available, prinomastat appears to inhibit their activity more effectively than marimastat in fluorogenic enzymatic assays. Our findings suggest that marimastat may have a minimal influence on the metalloprotease activities of cobra venoms. Limited research has tested these two inhibitors together on cobra venoms due to the lack of substantial metalloprotease activities in these venoms. Findings from one study indicated that marimastat and batimastat can inhibit the haemorrhagic and proteolytic activities of *E. ocellatus* venom [16]. Another study revealed that marimastat can block the metalloprotease activity of *D. russelii* venom [17]. Several recent studies have reported the impact of marimastat in rescuing mice from venom-induced lethality [12,17].

The caseinolytic assay is widely used to measure the proteolytic activities of venoms [18]. This assay quantifies the collective proteolytic activities of both metalloproteases and serine proteases [13]. Due to the abundance of these proteolytic enzymes in viper venoms, they often exhibit higher caseinolytic activities than elapid venoms, which typically contain a lower quantity of these enzymes [13,14]. Our findings from caseinolytic assays indicate that viper and cobra venoms demonstrate varying levels of proteolytic activities. Both marimastat and prinomastat inhibited the proteolytic activities of cobra and viper venoms, although prinomastat is slightly more effective in some cobra venoms. Similarly, both compounds were able to block the protease activities of nearly all viper venoms. Results from the fibrinogenolytic activities of cobra and viper venoms revealed that all viper venoms, except *D. russelii* (starts to degrade at 24 h), could degrade fibrinogen, specifically the α chain. A few of them began to digest the β chain after a few hours. Among all viper venoms, *B. arietans* exhibited a minimal but delayed effect on the γ chain. Therefore, all viper venoms were able to cleave the α and, in some instances, the β chains of fibrinogen, with little effect on the γ chain. These data align with the previously reported results for viper venoms [19,20,21,22]. Marimastat and prinomastat mostly blocked the fibrinolytic activities of the viper venoms within the first hour; however, after that, even samples with inhibitors began to digest the α-chain and this could be due to the high venom activity and limited amount of inhibitors. The only exceptions where both inhibitors functioned effectively during the 24-h incubation period were with *C. atrox* and *B. gabonica*. Notably, the fibrinogenolytic activities are also attributed to viper venom serine proteases, which often possess thrombin-like activities [23,24,25,26]. Therefore, these inhibitors are unlikely to suppress all the fibrinogenolytic activities induced by viper venoms. Similarly, all cobra venoms, except *N. sputatrix*, exhibited fibrinogenolytic activity specifically on the α chain, albeit to a lesser extent than viper venoms. Among cobra venoms, *N. nivea* and *N. atra* demonstrated the highest fibrinogenolytic activity. Both marimastat and prinomastat inhibited the fibrinogenolytic activities of all cobra venoms at all time points in a consistent manner. These results suggested that marimastat may not effectively inhibit the metalloprotease activities of cobra venoms in fluorogenic assays, although they might work similarly with natural substrates. Our findings are consistent with a previous study that reported the ability of *Naja* sp. venom to degrade the α chain of fibrinogen [27]. Another study, which tested the fibrinogenolytic activity of an isolated serine protease from *B. arietans* venom, showed that increasing the concentration of the enzyme enabled the cleavage of all α, β, and γ chains [28]. In addition, another study reported that *M. xanthina* venom could degrade the α and the β chains while the γ chain remained stable [29].

In conclusion, our findings demonstrate that different cobra venoms may exhibit varying sensitivities to marimastat and prinomastat. Our results from the metalloprotease assay indicate that marimastat may be less effective against cobra venoms than prinomastat, which could be due to the different types of metalloproteases present or other factors associated with cobra venoms themselves. In contrast, the data from caseinolytic assays show that both marimastat and prinomastat have nearly the same effect across almost all cobra venoms, except in *N. sputatrix* and *N. atra*, where prinomastat significantly inhibits more than marimastat. Furthermore, both inhibitors completely inhibit the fibrinolytic activities of all cobra venoms. Thus, the lower apparent efficacy of marimastat in the metalloprotease assay may have been influenced by factors inherent to the substrate (e.g., DQ-gelatin); however, this remains a hypothesis requiring further validation. Based on these in vitro findings, marimastat may not be an optimal choice as a metalloprotease inhibitor for venoms that possess very low levels of metalloprotease activity. Therefore, researchers should exercise greater caution when selecting an inhibitor for their experiments and prefer prinomastat over marimastat for venoms with low metalloprotease activity.

In the future, it would be worthwhile to attempt this experiment in an in vivo model by injecting all these venoms with each inhibitor into the muscles to ascertain whether these inhibitors can prevent muscle damage and lethality, and if so, to what extent. New findings and developments in the field might enable us to design more effective and selective therapeutic strategies, ultimately reducing the morbidity and mortality associated with snakebite envenomings.

## 4. Materials and Methods

### 4.1. Venoms Used

Snake venoms were obtained as lyophilised powders from various companies. Venoms of *N. naja*, *N. atra*, *N. nigricolis*, *N. sputatrix*, *Bothrops asper*, *C. oreganus*, and *C. basilicus* were purchased from Venomtech Ltd., Sandwich, UK. Venoms of *N. melanoleuca*, *N. annulifera*, and *N. nivea* were acquired from Latoxan, Valence, France. Venoms from *C. rhombeatus*, *E. carinatus*, *M. xanthina*, *A. c. laticinctus*, *T. stejnegeri*, and *Bitis arietans* were sourced from Sigma Aldrich, Poole, UK. Venoms of *D. russelli* and *C. atrox* were obtained from the Kentucky Zoo. The venom from *B. gabonica* was procured from the Liverpool School of Tropical Medicine, Liverpool, UK. The required concentrations of each venom were prepared by dissolving it in a phosphate-buffered saline (PBS). For long-term storage, the venoms were maintained as small aliquots at −80 °C, and prior to use, they were transferred to −20 °C.

### 4.2. Metalloprotease Assay

The metalloprotease activity of the venoms was measured using DQ-gelatin (ThermoFisher Scientific, Abingdon, UK) as a fluorogenic substrate. Concentrations of 100 µg/mL of various cobra venoms and 50 µg/mL of various viper venoms (in the presence and absence of different concentrations of marimastat and prinomastat (Sigma Aldrich, Poole, UK)) were added to a black 96-well plate. A final reaction volume of 100 µL was prepared with PBS. After 10 min of incubation at room temperature, two µg of DQ-gelatin were added to each well. The levels of fluorescence were measured at different time points using an excitation wavelength of 485 nm and an emission wavelength of 520 nm in a Fluostar Optima (BMG Labtech, Ortenberg, Germany) spectrofluorometer.

### 4.3. Fibrinogenolytic Assay

Fibrinogenolysis is a toxic effect caused by venom metallo and serine proteases [30]. This assay was conducted to demonstrate the ability of the venoms to hydrolyse fibrinogen chains. 100 μg/mL elapid venoms and 50 μg/mL viper venoms (with and without inhibitors) were mixed with fibrinogen (1 mg/mL) (Sigma Aldrich, UK) to achieve a final volume of 200 μL. Samples were then incubated at 37 °C for various time points. 30 μL of each sample was taken at 1, 3, 6, and 24 h and immediately mixed with 30 μL of 2× reducing sample treatment buffer (2× RSTB) before being frozen at −20 °C. Samples were boiled at 90 °C for 10 min prior to loading on the wells and analysing using sodium dodecyl sulfate-polyacrylamide gel electrophoresis (SDS-PAGE).

### 4.4. SDS-PAGE

For gel electrophoresis, 10-well 12% SDS-PAGE gels were hand-cast using the following method: resolving gel comprising 14 mL 30% (*w*/*v*) Acrylamide–bisacrylamide mix, 4.38 mL resolving gel buffer (3 M Tris-HCl, pH 8.8), 350 µL 10% (*w*/*v*) SDS, 1.75 mL 1.5% (*w*/*v*) ammonium persulfate, 14.525 mL water, and 34 µL tetra-methyl-ethylene-diamine (TEMED); stacking gel included 1.35 mL 30% (*w*/*v*) Acrylamide–bisacrylamide mix, 2.5 mL stacking gel buffer (0.5 M Tris-HCl, pH 6.8), 100 µL 10% (*w*/*v*) SDS, 500 µL 1.5% (*w*/*v*) ammonium persulfate (APS), 5.75 mL water, and 8 µL tetra-methyl-ethylene-diamine (TEMED). To produce protein profiles of crude venoms, 60 µg of each venom was mixed with 2× RSTB [40% (*w*/*v*) SDS, 10% (*v*/*v*) β-mercaptoethanol, 20% (*v*/*v*) glycerol and 10% stacking gel buffer in nano pure water and a trace amount of bromophenol blue as a tracking dye) and heated for 10 min at 90 °C. Thereafter, the samples were vortexed and loaded onto the gel alongside 5 µL of Precision Plus Protein Standards Dual Colour (Bio-Rad, Watford, UK). All samples were then run at 70 volts for 2 h using a Mini-PROTEAN Electrophoresis System (Bio-Rad, UK). The resulting gels were stained with Coomassie blue [0.1% (*w*/*v*) Coomassie brilliant blue in 40% methanol and 10% acetic acid] for 2 h and de-stained with a de-staining solution [10% (*v*/*v*) methanol and 10% (*v*/*v*) acetic acid] for 2 h on a rocker.

### 4.5. Caseinolytic Assay

The caseinolytic activity was measured using azocasein as a substrate as previously described [31]. To prepare the substrate solution, 5 mg of azocasein was diluted in 1 mL of buffer containing 50 mM Tris-HCl at pH 8.0. Each of the venoms was then dissolved in Tris-HCl buffer to a final concentration of 1 mg/mL. To measure the caseinolytic activity, 10 µL (20 µg) of venom was mixed with 90 µL of the substrate solution. All samples were incubated for 90 min at 37 °C. Following incubation, 200 µL of 5% (*v*/*v*) TCA was added to each sample, and then the samples were centrifuged for 5 min at 8000 rpm at room temperature. 150 µL of the supernatant were then added to a 96-well microplate, and 150 µL of NaOH (0.5 M) was subsequently added. The absorbance of each well was then read at 440 nm using spectrofluorimetry. For blank, Tris-HCl was used without venom.

### 4.6. Molecular Docking Analysis

To predict the inhibitory potential of marimastat and prinomastat, we selected metalloproteases from a viper and an elapid venom. The structure of VAP2 from *Crotalus atrox* venom (PDB ID: 2DW0; apo form without co-crystallised inhibitor) was used to define the catalytic Zn^2+^ site and docking grid. For reference to a hydroxamate inhibitor (e.g., GM6001/Ilomastat, ligand GM6), metal-coordinating geometry was imposed during docking [32]. The elapid venom metalloprotease from the venom of *Naja atra* structure of the ADAMalysin family lacks a co-crystallised ligand. Therefore, the zinc ion (PDB ID: 3K7N) was used as a reference [33]. Before docking, proteins were prepared using the Protein Preparation Wizard tool (version 2021-3) included in Maestro. Water (beyond 5 Å) was removed from the ligand structure, leaving the calcium and zinc metals intact. Hydrogens were then added, and ionisation states were calculated at pH 7.4 [34]. The proteins were energetically minimised with the OPLS4 force field. The centre of the boxes was localised using the crystallised ligand in the structure of VAP2. In contrast, in the structure of elapid snake venom metalloprotease a zinc ion was at the centre. Molecular docking simulations were performed for the structure of VAP2, with the outer edge of the grid set at 44 Å, and for the structure of the elapid metalloprotease, at 54 Å. For the docking simulations, the standard precision function of Glide [35], along with the ten best-coupled ligand pose solutions, were further subjected to post-processing and rescoring by calculating the binding free energy (ΔGbind) using the molecular mechanics generalised Born surface area (MM-GBSA) protocol in Prime [36]. The best complexes, based on their interactions and ΔGbind, underwent 25 ns of equilibrium molecular dynamics (MD) simulations, each using Desmond software (version 2021-3) and the OPLS4 force field [37]. Following this, 1 μs of production molecular dynamics was performed for each complex. To prepare both systems, the complexes were dissolved in pre-equilibrated water molecules with a single point charge in a periodic-boundary condition box. Neutralisation of the systems was carried out by adding Na^+^ or Cl^−^ counterions and, to simulate physiological conditions, a final concentration of 0.15 M NaCl was established. Each system was relaxed using the predetermined Desmond relaxation protocol and then equilibrated for 25 ns, with the NPT ensemble set at 1 atm and 300 K. A spring constant of 5.0 kcal mol^−1^ Å^−2^ was applied to the ligand, backbone, and ions. The final equilibrium MD framework was subsequently used to conduct 1 μs production MD under the same conditions as described above, with constraints on the secondary structure and ions.

### 4.7. Statistical Analysis

GraphPad Prism (version 7.0, GraphPad Inc., San Diego, CA, USA) was used to perform ordinary one-way ANOVA analysis and to assess statistical significance, followed by Fisher’s LSD test. The data are presented as mean ± SD. SPSS Statistics package (version 29.0, IBM Corp., Armonk, NY, USA) was used to perform two-way ANOVA analysis to evaluate the effects of species and inhibitor treatment. Normality and homogeneity of variances were verified using Shapiro–Wilk and Levene’s tests, respectively. Post-hoc comparisons were conducted using Tukey’s test, and data are presented as mean ± SEM.

## Data Availability

The original contributions presented in this study are included in the article/Supplementary Material. Further inquiries can be directed to the corresponding authors.

## Supplementary Materials

The following supporting information can be downloaded at: https://www.mdpi.com/article/10.3390/toxins17120571/s1. Figure S1: Fibrinogenolytic activity of cobra venoms showing the degradation products over time.
