# Supplementary figures and images for: Differential Effects of Marimastat and Prinomastat on the Metalloprotease Activity of Various Snake Venoms
Source: Toxins (Basel). 2025 Nov 26;17(12):571. doi: 10.3390/toxins17120571 (PMC12737354; doi:10.3390/toxins17120571)

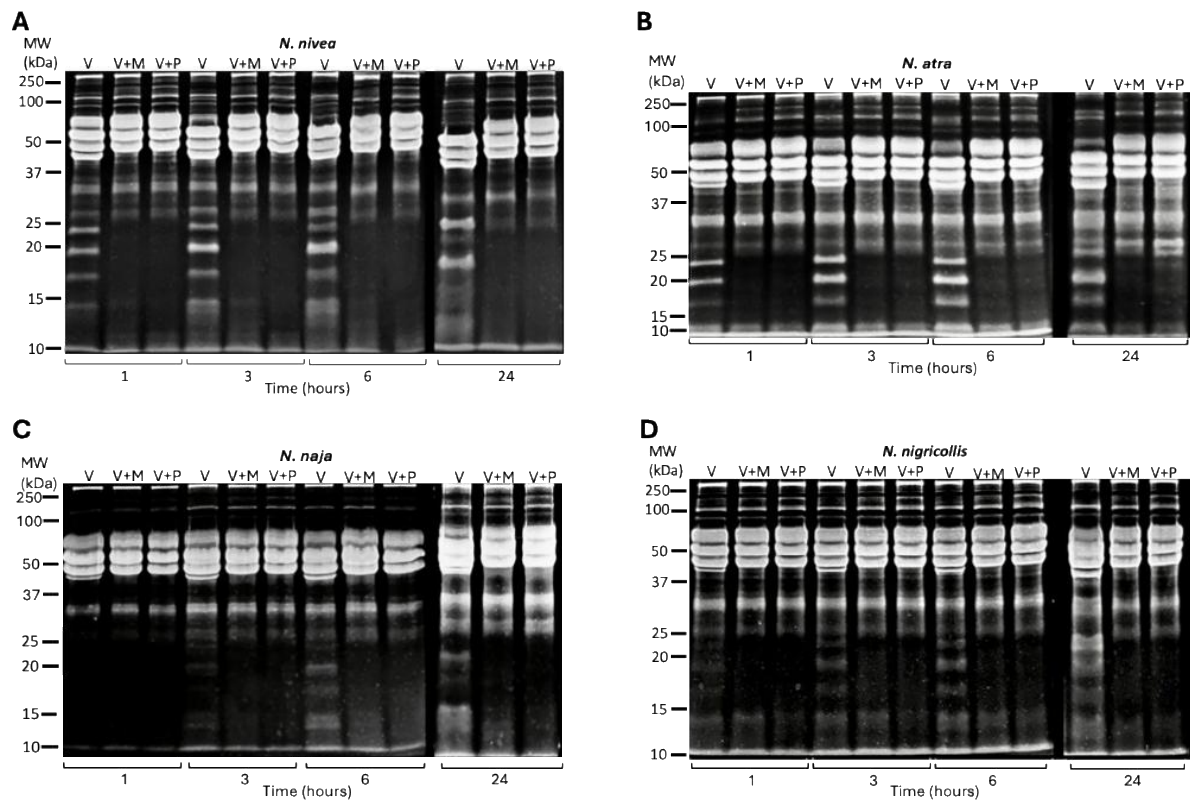

Supplementary File S1: Fibrinogenolytic activity of cobra venoms.

Supplement: Supplementary file 1 [file toxins-17-00571-s001.zip › toxins-3917739-supplementary.pdf]
